# Polyoxazoline multivalently conjugated with indocyanine green for sensitive *in vivo* photoacoustic imaging of tumors

**DOI:** 10.1038/srep33798

**Published:** 2016-09-26

**Authors:** Kengo Kanazaki, Kohei Sano, Akira Makino, Tsutomu Homma, Masahiro Ono, Hideo Saji

**Affiliations:** 1 Department of Patho-Functional Bioanalysis Graduate School of Pharmaceutical Sciences, Kyoto University, 46-29 Yoshida Shimoadachi-cho, Sakyo-ku, Kyoto, 606-8501, Japan; 2 Medical Imaging Project, Corporate R&D Headquarters, Canon Inc., 3-30-2 Shimomaruko, Ohta-ku, Tokyo, 146-8501, Japan; 3 Kyoto University Hospital, 54 Kawaharacho, Shogoin, Sakyo-ku, Kyoto, 606-8507, Japan; 4 Biomedical Imaging Research Center, University of Fukui, 23-3 Matsuokashimoaizuki, Eiheiji-cho, Yoshida-gun, Fukui, 910-1193, Japan

## Abstract

Photoacoustic imaging, which enables high-resolution imaging in deep tissues, has lately attracted considerable attention. For tumor imaging, photoacoustic probes have been proposed to enhance the photoacoustic effect to improve detection sensitivity. Here, we evaluated the feasibility of using a biocompatible hydrophilic polymer, polyoxazoline, conjugated with indocyanine green (ICG) as a tumor-targeted photoacoustic probe *via* enhanced permeability and retention effect. ICG molecules were multivalently conjugated to partially hydrolyzed polyoxazoline, thereby serving as highly sensitive photoacoustic probes. Interestingly, loading multiple ICG molecules to polyoxazoline significantly enhanced photoacoustic signal intensity under the same ICG concentration. *In vivo* biodistribution studies using tumor bearing mice demonstrated that 5% hydrolyzed polyoxazoline (50 kDa) conjugated with ICG (ICG/polyoxazoline = 7.8), P14-ICG7.8, showed relatively high tumor accumulation (9.4%ID/g), resulting in delivery of the highest dose of ICG among the probes tested. P14-ICG7.8 enabled clear visualization of the tumor regions by photoacoustic imaging 24 h after administration; the photoacoustic signal increased in proportion with the injected dose. In addition, the signal intensity in blood vessels in the photoacoustic images did not show much change, which was attributed to the high tumor-to-blood ratios of P14-ICG7.8. These results suggest that polyoxazoline-ICG would serve as a robust probe for sensitive photoacoustic tumor imaging.

Optical technologies have greatly advanced in the past decades and have been applied for several medical imaging techniques, including optical coherent tomography, fluorescence imaging, and photoacoustic (PA) imaging^1^. Among them, PA imaging, which detects ultrasound thermoelastically generated from the optical absorbers irradiated with pulsed laser, has emerged as an attractive modality due to sensitive and high resolution imaging at a depth of several centimeters *in vivo*^2^,^3^. Currently, various PA signal-enhancing contrast agents (PA probes), including metallic nanoparticles^4^,^5^ and fluorescence dyes^6^,^7^,^8^ have been reported to aid in the diagnosis of tumors. However, these probes still remain in pre-clinical and clinical trials and are not in clinical use.

Because of its biocompatibility^9^, PA probes labeled with indocyanine green (ICG), a US Food and Drug Administration (FDA)-approved near-infrared fluorescence dye, have been investigated^10^. In our previous studies, the feasibility of using proteins and polyethylene glycol (PEG) conjugated with ICG for tumor-targeted PA probes has been demonstrated^11^,^12^,^13^. PEG with large molecular weight (>20 kDa) accumulated in the tumor *via* enhanced permeability and retention (EPR) effect^14^,^15^, and visualization of tumors was achieved in PA imaging. However, the prolonged half-life of PEG in the blood lowered tumor-to-blood ratios, resulting in PA tumor images with low contrast (brightly enhanced blood vessels were seen). Furthermore, multiple ICG molecules could not be conjugated to PEG, because only the terminal of polymer could be used to conjugate (*e.g.* ICG/PEG = 1 in our previous study^12^). To overcome this problem, we selected polyoxazoline (POZ) as a novel carrier of PA signal emitters.

POZ, a hydrophilic polymer composed of *N*-acyl ethyleneimine with low toxicity *in vitro* and *in vivo*^16^,^17^, could be synthesized *via* living cationic ring-opening polymerization^18^,^19^. POZ could be characterized by rapid clearance from the circulation^20^ and was utilized as a modulator of drug delivery carriers, including nanoparticles^21^,^22^ and proteins^23^,^24^. However, no study has utilized POZ itself as a carrier of diagnostic probes. Therefore, in this study, the feasibility of using POZ labeled with ICG as a contrast agent for PA tumor imaging *via* the EPR effect was evaluated. Furthermore, we deduced that multiple ICG molecules could be conjugated with POZ through secondary amino groups introduced by hydrolyzing acyl groups of POZ^25^,^26^.

In this report, as shown in Fig. 1, we initially determined optimal molecular weight assuring tumor uptake *via* the EPR effect (Fig. 1A). Second, after partial hydrolysis of propionyl groups in POZ, the effect of hydrolysis ratio on tumor uptake was assessed (Fig. 1B). Third, we verified the relationship between the amount of ICG conjugated to POZ and tumor accumulation (Fig. 1C). Finally, we evaluated the feasibility of POZ multivalently conjugated with ICG (POZ-ICG) as a tumor-targeted probe for sensitive *in vivo* PA imaging.

## Results

### Optimization of Molecular Weight of POZ for Accumulation in Tumors *via* the EPR Effect

In order to determine the optimal molecular weight of POZ taken up by the tumor *via* the EPR effect, a series of POZ molecules conjugated with an ICG molecule were synthesized according to Supplementary Fig. S1A or S1B. The POZ polymer was synthesized *via* living cationic polymerization using microwave irradiation, and the molecular weight of the obtained POZ and number of ICG molecules conjugated to POZ are summarized in Table 1. The chemical purity of POZ-ICG was >95%, as defined by size-exclusion chromatography (Supplementary Fig. S2).

**Figure 2:**
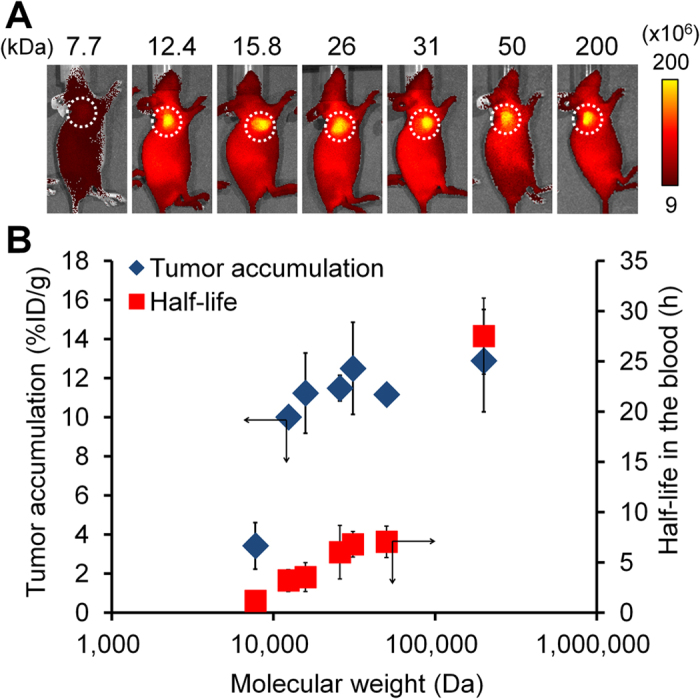
*In vivo* fluorescent measurement of POZ-ICG derivatives with different molecular weights. (**A**) *In vivo* fluorescence imaging of tumor-bearing mice administered with POZ-ICG (left to right; P1-ICG1.1, P2-ICG1.1, P3-ICG0.9, P4-ICG0.7, P5-ICG0.8, P6-ICG1.0, and P7-ICG1.1). Dotted circles indicated the tumor regions. Scale bar units: photons/sec/cm^2^/steradian. (**B**) Tumor accumulation (%ID/g) (blue) and half-life in the blood (h) (red) of POZ-ICG.

POZ-ICG derivatives were administered into the colon 26 tumor-bearing mice, and the tumor uptake and blood clearance were investigated (Fig. 2). A high tumor uptake (11–13% injected dose (ID)/g) was observed when the molecular weight of POZ was 15.8 kDa and more, indicating that this range of molecular weight is preferable for efficient tumor uptake *via* the EPR effect. The half-life in the blood was prolonged as the molecular weight of POZ-ICG increased. However, tumor-to-blood (T/B) ratios at 24 h after injection were more than three when the molecular weight of POZ was less than 50 kDa, which was significantly higher than those of PEG conjugated with ICG (PEG-ICG) we have previously examined (T/B ratios; 1.2 and 0.7 at 24 h post-injection for PEG-ICG (20 kDa) and PEG-ICG (40 kDa), respectively). Therefore, the potential of POZ-ICG as a PA probe for tumor diagnosis was demonstrated. In order to ensure high level of tumor uptake and tumor-to-blood ratios, we selected POZ with molecular weight of 25 and 50 kDa for subsequent experiments.

### Evaluation of Hydrolyzed POZ Conjugated with Multiple ICG Molecules

For the preparation of POZ conjugated with multiple ICG molecules, acyl groups of POZ were partially hydrolyzed by refluxing with hydrochloric acid (Supplementary Fig. S1B), resulting in introduction of secondary amino groups (2.5, 5, 10, 15, and 20% hydrolysis ratio) (Supplementary Fig. S3). The p*K*a values and electric conductivity of each hydrolyzed POZ are summarized in Table 2. All of the POZ derivatives exhibited p*K*a values of approximately 10. Moreover, as the hydrolysis ratio of POZ increased, electric conductivity decreased due to consumption of protons.

**Figure 3:**
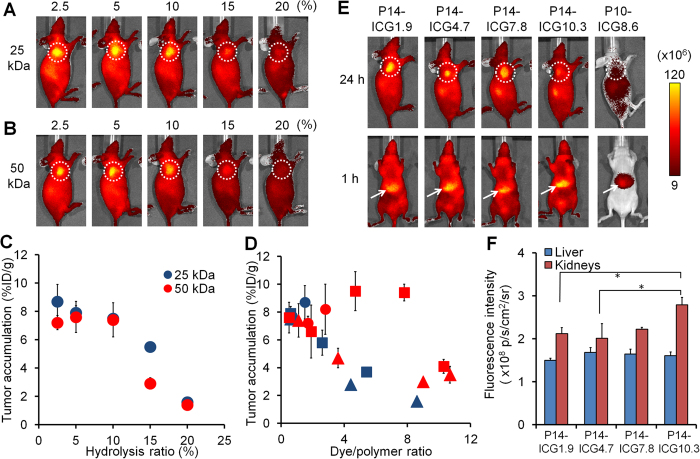
Influence of POZ hydrolysis ratios and numbers of ICG molecules on *in vivo* biodistribution. (**A**) *In vivo* fluorescence imaging of tumor-bearing mice administered with POZ-ICG (25 kDa) (left to right; P8-ICG1.5, P9-ICG0.6, P10-ICG0.6, P11-ICG0.7, and P12-ICG1.9, as shown in Table 2). (**B**) *In vivo* fluorescence imaging of tumor-bearing mice administered with POZ-ICG (50 kDa) (left to right; P13-ICG1.7, P14-ICG0.5, P15-ICG1.0, P16-ICG1.1 and P17-ICG1.9, as shown in Table 2). Dotted circles indicated tumor regions. Scale bar units: photons/sec/cm^2^/steradian. (**C**) Tumor accumulation of POZ-ICG (%ID/g) (Blue; 25 kDa, Red; 50 kDa). (**D**) Tumor accumulation of POZ-ICG with molecular weight of 25 kDa (blue) and 50 kDa (red). Circles, squares, and triangles indicate hydrolysis ratios of 2.5, 5, and 10%, respectively. (**E**) *In vivo* fluorescence images of tumor bearing mice at 24 h (upper) and 1 h (lower) after administration of POZ-ICG. Left to right; P14-ICG1.9, P14-ICG4.7, P14-ICG7.8, P14-ICG10.3, and P10-ICG8.6. Dotted circles and white arrows indicate the tumor and liver regions, respectively. (**F**) Fluorescence intensity of liver and kidneys isolated from the mice at 24 h after administration of POZ-ICG (*P < 0.05).

Prior to conjugation of multiple ICG molecules into POZ, we evaluated the influence of POZ hydrolysis ratios (percentage of secondary amino groups per POZ polymer) on *in vivo* biodistribution. The accumulation of the POZ series labeled with ICG (ICG/POZ = 0.5–1.9) (Table 2) in the tumor was investigated using colon 26 tumor-bearing mice. Independent of molecular weights of POZ (25 and 50 kDa), the tumor uptake was reduced as the hydrolysis ratios were increased (Fig. 3A–C). Moreover, increase in hydrolysis ratios was accompanied by rapid clearance of POZ from the blood, and the half-life of POZ in the blood was correlated with electrical conductivity, as shown in Table 2.

Since high tumor uptake (more than 7%ID/g) was ensured in the hydrolysis ratios of 2.5–10% (Fig. 3C), conjugation of multiple ICG molecules was undertaken for POZ-ICG (25 and 50 kDa, 2.5–10% hydrolysis ratio), as shown in Table 3, and thereafter, *in vivo* biodistribution in colon 26 tumor-bearing mice was investigated. In the case of 2.5% hydrolysis ratio, as the number of ICG molecules conjugated to POZ increased, tumor accumulation slightly increased for both molecular weights (25 and 50 kDa) (Fig. 3D). However, a large amount of ICG could not be loaded due to limited reaction sites. Regarding 5% hydrolyzed POZ-ICG (50 kDa), tumor uptake was highly sustained when the number of ICG molecules introduced was up to 7.8. However, the introduction of excess ICG molecules severely compromised the delivery of POZ-ICG into tumor tissues. The other probes (5 and 10% hydrolyzed POZ-ICG (25 kDa) and 10% hydrolyzed POZ-ICG (50 kDa)) demonstrated a decrease in tumor accumulation as the number of ICG increases. From the results of *in vivo* fluorescence imaging, POZ-ICG loaded with excess ICG molecules preferred hepatic uptake early post-injection (Fig. 3E). In order to elucidate this mechanism, the binding affinities of POZ-ICG derivatives (50 kDa, 5% hydrolyzed) to serum albumin were measured. When the number of ICG molecules loaded to POZ increased (1.9, 4.7, 7.8, and 10.3), the binding affinity to serum albumin (*K*_b_ values) also increased (3.0 ± 0.1, 3.5 ± 0.2, 8.7 ± 1.1, and 13.0 ± 0.4 M^−1^, respectively).

To investigate the probe accumulation in normal tissues, the fluorescence intensity in the liver and kidneys isolated from mice administered with POZ-ICG (50 kDa, hydrolysis ratio 5%, ICG/POZ = 1.9–10.3), which show high tumor accumulation, was measured. At 24 h after administration, while the fluorescence intensity in the liver was almost same among all of POZ-ICG, P14-ICG10.3 showed the highest intensity in the kidneys (Fig. 3F). The probe concentration in the blood at 24 h after administration decreased with increase in the number of conjugated ICG molecules (Supplementary Table S1). Therefore, tumor-to-blood ratios of all multivalently ICG-conjugated POZ were greater than 2.

### *In Vitro* Fluorescence and PA Measurement

**Figure 4:**
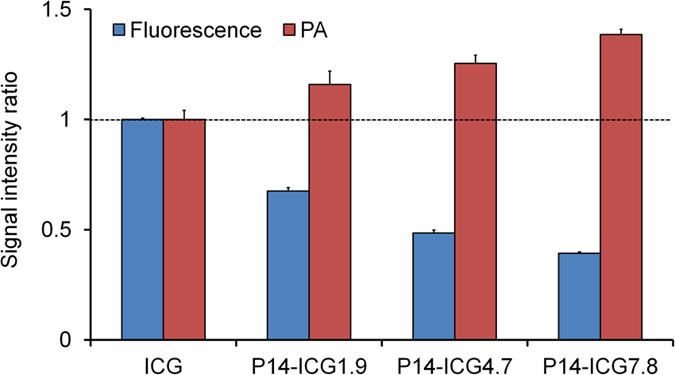
Signal intensity ratio of POZ-ICG (P14-ICG1.9, P14-ICG4.7, and P14-ICG7.8) and ICG. Blue: fluorescence intensity ratio, red: PA signal intensity ratio. Data were normalized by the signal intensity of ICG.

Owing to high tumor accumulation observed in the *in vivo* biodistribution study, we selected P14 (50 kDa, 5% hydrolysis ratio) conjugated with multiple ICG molecules (1.9, 4.7, and 7.8) for the subsequent experiments. First, the fluorescence intensities of each compound and ICG were measured. The fluorescence intensity of POZ-ICG was decreased, accompanied by an increase in the number of ICG molecules loaded (Fig. 4). Subsequently, the PA signal intensity was measured. Interestingly, the PA signal intensity of POZ-ICG was significantly increased with increase in the number of conjugated ICG molecules (39% increase for P14-ICG7.8) (Fig. 4) when compared under the same ICG concentration.

### *In Vivo* Photoacoustic Imaging Study

**Figure 5:**
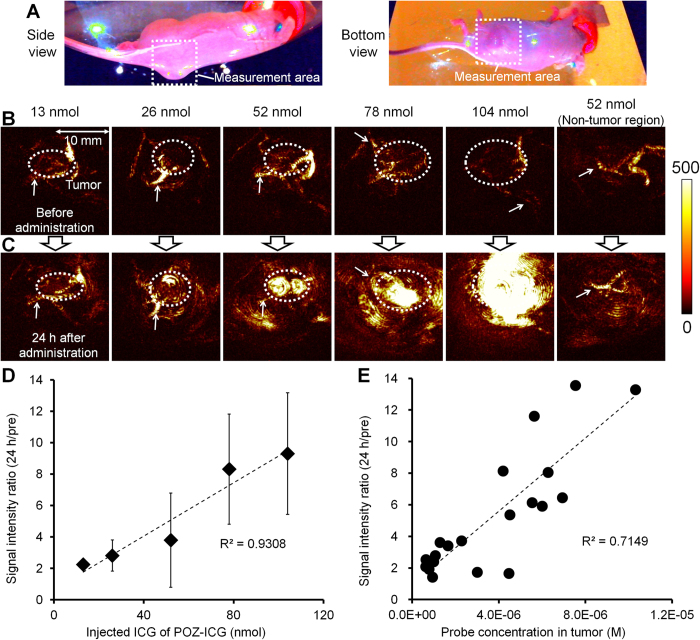
*In vivo* PA imaging of tumor-bearing mice administered P14-ICG7.8. Photographs of tumor-bearing mice placed in the PA imaging device. (**A**) The square region indicates the measurement area. (**B**,**C**) PA images (view from the bottom) before (**B**) and at 24 h after (**C**) probe administration (Left to right: tumor regions injected with 13, 26, 52, 78, and 104 nmol ICG and non-tumor regions (thighs) injected with 52 nmol ICG). Dotted circles and arrows indicated the tumor regions and blood vessels, respectively. Scale bar units: arbitrary unit. (**D**) Plot of PA signal intensity ratio (24 h/pre) vs. injected dose of P14-ICG7.8. (**E**) Plot of PA signal intensity ratio (24 h/pre) vs. P14-ICG7.8 concentration in isolated tumors.

Finally, we evaluated the feasibility of POZ-ICG as a tumor-targeted PA imaging probe. P14-ICG7.8 was investigated for further *in vivo* PA imaging studies because of the high tumor accumulation, high tumor-to-background ratio (Fig. 3), and high PA signal intensity (Fig. 4). *In vivo* PA imaging of tumor region (2 × 2 × 2 cm) at the mouse thigh was performed (Fig. 5A). Before probe injection, only blood vessels were observed (Fig. 5B, supplementary video S1). At 24 h after administration with P14-ICG7.8 (13 nmol or more), the tumor regions were clearly visualized and PA signal intensity in blood vessels was hardly changed (Fig. 5C, supplementary video S2). The PA signal intensity in the tumor region was significantly higher than that in the non-tumor region when P14-ICG7.8 (52 nmol ICG) was administered (4.7 ± 2.5 × 10^5^ vs 1.2 ± 0.6 × 10^5^ (a.u.) for tumor vs non-tumor) (Fig. 5B,C). The increase in PA signal generated by P14-ICG7.8 in the tumor region had a good linearity relationship with the injected dose in the range of measurement (124 ± 28, 181 ± 99, 279 ± 300, 731 ± 351%, and 830 ± 387% increase compared to pre-injection for 13, 26, 52, 78, and 104 nmol ICG, respectively) (Fig. 5D). Furthermore, PA signal intensity was also in proportion with ICG concentration in isolated tumors (Fig. 5E).

## Discussion

In this study, we synthesized POZ conjugated with multiple ICG molecules and optimized its molecular weight, hydrolysis ratio of acyl groups, and number of ICG loaded to POZ as a tumor-targeted PA imaging probe.

Based on tumor uptake and tumor-to-background ratios, optimal molecular weight was determined to be 25–50 kDa. When POZ was hydrolyzed, the electric conductivity decreased as the hydrolysis ratio increased (Table 2). This result suggested that hydrolyzed POZ would be positively charged *in vivo* and that this charge could increase depending on the hydrolysis ratio. In *in vivo* biodistribution study, the half-life in the blood of POZ-ICG (hydrolysis ratio: 2.5–20%) decreased as electrical conductivity decreased as shown in Table 2, suggesting that rapid clearance is related to the positive charge of POZ; especially high hepatic uptake of P12-ICG1.9 and P17-ICG1.9 was noted early after injection by *in vivo* fluorescence imaging at 1 h after administration (Supplementary Fig. S4). This result was supported by previous reports wherein nanoparticles with positive charge showed shortened half-life in the blood^27^,^28^.

As we previously reported, pharmacokinetics of PEG-ICG were changed by interaction between ICG and serum albumin^12^. Therefore, we measured the binding affinity (*K*_b_ value) of POZ-ICG (50 kDa, 5% hydrolysis). *K*_b_ values increased as the number of ICG increased, suggesting that POZ-ICG with high *K*_b_ value accumulated in the liver not only *via* being albumin-probe macro-conjugates but also by orienting ICG to the liver. On the other hand, in some specific cases (i.e. 50 kDa, hydrolysis ratio 5%, ICG/POZ = 1.9–7.8), conjugation of ICG to POZ improved tumor uptake probably due to lowering hydrolysis ratios. Furthermore, the influence of hydrolysis ratio or number of ICG molecules loaded on tumor uptake was marked for low molecular weight of POZ (25 kDa).

In *in vitro* measurement, the fluorescence intensity of POZ-ICG was decreased as the number of ICG increased (Fig. 4), suggesting that fluorescence signal of ICG conjugated to POZ at high density was partially quenched by intermolecular interaction, as reported previously^29^. On the other hand, the PA signal intensity was increased with increase in the number of ICG (Fig. 4), indicating that the fluorescence quenching energy of ICG was used to generate the PA signal. In addition, when ICG molecules were conjugated with POZ at high density, the thermal energy might be generated locally, which could improve its conversion efficiency to the acoustic signal^30^,^31^.

We have previously evaluated the feasibility of biocompatible and FDA-approved polymer (PEG and human serum albumin (HSA)) conjugated with ICG as PA probes^11^,^12^. POZ-ICG maintained high tumor accumulation (9.4%ID/g at 24 h post-injection for P14-ICG7.8) even though large number of ICG molecules was loaded unlike ICG-conjugated HSA (HSA-ICG, ICG/HSA = 8.2) (2.1%ID/g at 24 h post-injection)^11^. Furthermore, POZ could be multivalently conjugated with ICG molecules compared with PEG-ICG (20 kDa, ICG/PEG = 1)^12^, and POZ-ICG showed high tumor-to-blood ratio without compromising tumor uptake. Although the tumor accumulation of P14-ICG7.8 was slightly less (9.4 ± 0.6 and 14.9 ± 1.2%ID/g at 24 h post-injection for P14-ICG7.8 and PEG-ICG, respectively), the PA signal intensity ratio (24 h post-injection/pre-injection) was comparable (8.3 ± 3.9 and 7.2 ± 2.1 for P14-ICG7.8 and PEG-ICG, respectively) when the same amount of ICG was injected. This was probably due to increase in PA signal per ICG molecules as shown in Fig. 4. These results demonstrated the benefits of POZ multivalently conjugated with ICG molecules for sensitive *in vivo* PA tumor imaging even if the lower dose of polymer was injected (≈10% compared with PEG). Furthermore, the tumor-to-blood ratios of P14-ICG7.8 were significantly higher than those of PEG-ICG (6.3 ± 1.0 and 1.2 ± 0.1 for P14-ICG7.8 and PEG-ICG, respectively). In fact, P14-ICG7.8 hardly changed the PA signal intensity in blood vessels while clearly visualizing tumor regions (Fig. 5B,C), thus, achieving high contrast tumor PA imaging. The biosafety of both ICG and POZ has been reported individually^9^,^17^. Furthermore, no noticeable side effect was observed in mice, suggesting low toxicity in the range of doses examined. Although the biosafety of POZ-ICG conjugate needs to be assessed in detail, the likelihood of smooth translation to the clinical study would be expected.

## Conclusions

In this study, we elucidated (i) a potential of POZ-ICG for PA tumor imaging utilizing tumor accumulation *via* the EPR effect and (ii) loading of multiple ICG molecules achieved sensitive *in vivo* PA tumor imaging. Given tumor accumulation and tumor-to-background (blood) ratios, optimal molecular weight was determined to be 25–50 kDa. Although the hydrolysis of propinyl groups in POZ partially compromised tumor accumulation due to increasing positive charge, 2.5–10% hydrolyzed POZ derivatives sustained their tumor uptake. Among contrast agents loaded with multiple ICG molecules, P14-ICG7.8 (5% hydrolyzed POZ (50 kDa) conjugated with ICG molecule of 7.8) exhibited high tumor uptake and background ratios. Furthermore, PA signals were significantly enhanced by loading multiple ICG molecules to POZ. Finally, P14-ICG7.8 clearly visualized the tumors with high contrast in the *in vivo* PA imaging study, suggesting the feasibility of POZ-ICG as a contrast agent for sensitive PA tumor imaging.

## Methods

### General

Proton nuclear magnetic resonance (^1^H NMR) spectra were obtained using a JEOL JNM-LM400 with CDCl_3_ as a solvent and tetramethylsilane as an internal standard. Coupling constants are reported in hertz. Gel permeation chromatography (GPC) was performed with a LC-20AD Shimadzu pump and a Shimadzu RID-10A refractive index detector equipped with a GPC column KD 804 (Showa Denko, Tokyo, Japan) with *N*,*N*-dimethylformamide (DMF) as the mobile phase at a flow rate of 0.5 mL/min. Weight-average molecular weight was determined by a calibration curve calculated using PEG standards (Agilent Technologies, Santa Clara, CA, USA).

### Materials

Methyl *p*-toluenesulfonate, ethylenediamine, and super dehydrated acetonitrile were purchased from Wako Pure Chemical Industries, Ltd. (Osaka, Japan). 2-Ethyl-2-oxazoline was purchased from Tokyo Chemical Industry Co., Ltd. (Tokyo, Japan). 1-Ethyl-3-(3-dimethylaminopropyl) carbodiimide hydrochloride (WSC) was purchased from Dojindo Molecular Technologies, Inc. (Kumamoto, Japan). 4-Dimethylaminopyridine (DMAP) and 2 mol/l-hydrochloric acid were purchased from Nacalai Tesque, Inc. (Kyoto, Japan). Poly(2-ethyl-2-oxazoline) (molecular weight: 25, 50, and 200 kDa) was purchased from Sigma-Aldrich Japan (Tokyo, Japan). 1H-Benz[e]indolium, 2-[7-[3-(5-carboxypentyl)-1,3-dihydro-1,1-dimethyl-2H-benz[e]indol-2-ylidene]-1,3,5-heptatrien-1-yl]-1,1-dimethyl-3-(4-sulfobutyl), inner salt (ICCA) was synthesized according to previously reported method^32^. All other chemicals of highest purity available were used.

### Cell Culture and Animal Model

A mouse rectal cancer cell line, colon 26, was purchased from Riken Bio Resource Center (Ibaraki, Japan). The cells were maintained according to previous report^11^.

Animal studies were conducted in accordance with the institutional guidelines of Kyoto University and the experimental procedures were approved by the Kyoto University Animal Care Committee. Tumor-bearing mice were prepared as reported previously^11^. Briefly, colon 26 (1 × 10^6^ cells) suspended in 50 μL PBS were inoculated subcutaneously in the shoulders or thighs of the mice. Experiments with tumor-bearing mice were performed 7 days after inoculation.

### Preparation of POZ-ICG

POZ (molecular weight: 7.7–31 kDa) was synthesized *via* a microwave irradiation method as previously reported^18^,^19^. Briefly, methyl *p*-toluenesulfonate (5 mg, 1 eq.) and 2-ethyl-2-oxazoline (1.1 g, 400 eq.) were dissolved in super dehydrated acetonitrile (0.5–3 mL) in a capped reaction tube and stirred for 14 min at 140 °C by microwave irradiation using the Discover system (CEM Co., Matthews, NC, USA). Subsequently, ethylenediamine (16 mg, 10 eq.) was added to the reaction solution and stirred for 7 min at 140 °C *via* microwave irradiation. After solvent evaporation, the resulting mixture was dissolved in 2 mL methanol and dialyzed against methanol with pre-treated regenerated cellulose (RC) membrane Spectra/Por^®^ 7 dialysis tubing (molecular weight cut-off: 3.5 kDa) (Spectrum Laboratories, Inc., Rancho Dominguez, CA, USA) for purification. The resulting polymer was analyzed by ^1^H NMR and GPC. ^1^H NMR (CDCl_3_): δ [ppm] = 3.3–3.6 (br, 4nH, NC*H*_2_C*H*_2_N), 2.2–2.5 (br, 2nH, C=OC*H*_2_), 1.0–1.2 (br, 3nH, C*H*_3_).

POZ (7.7–31 kDa) (50–200 mg, 1 eq.) was stirred with ICCA (10 mg, 2 eq.), WSC (2.5 mg, 2 eq.), and DMAP (1.6 mg, 2 eq.) in chloroform (2 mL) at r.t. for 24 h with shielding from light. After solvent evaporation, the resulting mixture was dissolved in methanol (2 mL) and dialyzed against methanol as mentioned above to remove unconjugated ICCA. The concentration of ICG was determined by measuring the absorption at 795 nm in the presence of 5% SDS with a UV-Vis NIR system (UV-1800, Shimadzu Co., Kyoto, Japan). To determine the chemical purity, the purified POZ-ICG (100 pmol ICG) was separated by PD-10 column.

### Preparation of Hydrolyzed POZ Conjugated with Multiple ICG Molecules

For preparation of POZ loaded with multiple ICG molecules, poly(2-ethyl-2-oxazoline) (molecular weight: 25, 50, 200 kDa) was hydrolyzed to introduce secondary amine groups according to a previous report^26^. Briefly, poly(2-ethyl-2-oxazoline) in distilled and deionized water (250 mg, 25 mg/mL) was mixed with hydrochloric acid (2 M, 0.1–0.8 mL) and refluxed in an oil bath for 24 h. After neutralization, the resulting solution was freeze-dried. Finally, after filtration of the resulting mixture dissolved in chloroform using celite, partly hydrolyzed POZ was obtained. ^1^H NMR (CDCl_3_): δ [ppm] = 3.3–3.6 (br, 4nH, C*H*_2_N(C=O)C*H*_2_), 2.6–2.9 (br, 4nH, C*H*_2_NHC*H*_2_), 2.2–2.5 (br, 2nH, C=OC*H*_2_), 1.0–1.2 (br, 3nH, C*H*_3_).

The hydrolysis ratio of hydrolyzed POZ was determined by ^1^H NMR and electric conductivity measurement. The electric conductivity of hydrolyzed POZ in 0.01 M hydrochloric acid (60 μM) was measured by LAQUAtwin COND (Horiba, Ltd., Kyoto, Japan). The p*K*a value of each hydrolyzed POZ was determined using acid-base titration as previously reported^33^. Briefly, hydrolyzed POZ (0.6 μmol amino group) was dissolved in ddH2O (1 mL) and mixed with aqueous sodium hydroxide (0.01 M, 60 μL) to deprotonize all the amino groups. Neutralizing titration using 0.001 M hydrochloric acid was performed with a 9618S-10D pH meter (Horiba, Ltd.).

Subsequently, hydrolyzed POZ was conjugated with ICCA. POZ (25, 50 and 200 kDa) (10 mg, 1 eq.) was stirred with ICCA (1–300 eq.), WSC (1–300 eq.), and DMAP (1–150 eq.) in chloroform (2 mL) at r.t. for 24 h with shielding from light. The resulting solution was purified as mentioned above, and the purity of hydrolyzed POZ-ICG was determined by size exclusion chromatography.

The binding affinities of POZ-ICG to albumin were measured using previously reported procedures^12^. Briefly, each POZ-ICG (0–100 μM) was incubated with bovine serum albumin (BSA) (4 μM) for 30 min for equilibration. The fluorescence intensity of tryptophan in BSA was measured using a spectrofluorophotometer (RF-6000, Shimadzu Co., ex/em 279/342 nm). The binding affinity of each compound to BSA was calculated from the Hill equation.

### Fluorescence Measurement of POZ-ICG

The accumulation of POZ-ICG in the tumor was measured as reported previously^11^,^12^. Briefly, POZ-ICG (13 nmol ICG in 100 μL PBS) was intravenously injected into the colon 26 tumor-bearing nude mice (n = 3). At 1 and 24 h after administration of POZ-ICG, whole-body fluorescence images were acquired using the IVIS Imaging System 200 (ex: 745 nm, em: 840 nm, exposure time: 1 sec). At 24 h after administration, the tumors were excised and homogenized by adding 1% triton-X aqueous solution. After centrifugation, the supernatant (2 μL) was mixed with DMSO (18 μL) to extract POZ-ICG, and the fluorescence intensity was measured. In the same way, the mouse blood (2 μL) was collected at 5, 15, and 30 min, and 1, 3, 6, and 24 h after administration, then the blood was mixed with a 1% triton-X aqueous solution (9 μL) and DMSO (9 μL), and the fluorescence intensity was measured. The concentration of each POZ-ICG (%ID/g) in the tumor and blood was calculated using a standard curve prepared from each POZ-ICG diluted with tumor homogenate and blood, respectively. Tumor-to-blood ratio was calculated as a ratio of concentration of POZ-ICG (%ID/g) in the tumor and blood. The half-life of POZ-ICG in the blood was calculated by GraphPad Prism software (GraphPad Prism software, Inc. La Jolla, CA, USA). At 24 h after administration, the liver and kidneys were resected and the fluorescence intensity was measured using the IVIS Imaging System 200 (ex: 745 nm, em: 840 nm, exposure time: 1 sec).

### *In Vitro* Fluorescence and PA Measurement

Fluorescence intensity of POZ-ICG in 50 mg/mL BSA aqueous solution (15 μM ICG) was measured (ex: 785 nm, em: 815 nm) with an Infinite^®^ 200 PRO plate reader (Tecan Japan Co., Ltd., Kanagawa, Japan).

The photoacoustic signal measurement system was assembled as previously reported^12^. The PA signal intensity of POZ-ICG in 50 mg/mL BSA aqueous solution (15 μM ICG) was normalized by both the pulsed light intensity and the sample concentration.

### *In Vivo* PA Imaging

*In vivo* PA imaging was performed with an Endra Life Sciences Nexus 128 instrument (Endra Inc., Ann Arbor, MI, USA) as previously reported^11^,^12^. POZ-ICG (13, 26, 52, 78, and 104 nmol ICG in 100 μL PBS) was intravenously injected into the colon 26 tumor-bearing mice (n = 4). PA images of tumor region and non-tumor region (thigh) were acquired (120 angles, 20 pulses/angle, 797 nm) before injection and at 24 h after injection. The tumor was positioned at the center of the PA imaging region to ensure that the same regions could be imaged before and after probe administration. The 2D and 3D PA images were described *via* OsiriX software and PA signal intensity was calculated from the value of data points in volume rendering images normalized with irradiated laser intensity.

### Statistical Analysis

Statistical significance among groups was identified using the two-tailed Student’s *t*-test. Data are presented as the mean ± standard deviation. *P* values of less than 0.05 were considered statistically significant.

## Additional Information

**How to cite this article**: Kanazaki, K. *et al*. Polyoxazoline multivalently conjugated with indocyanine green for sensitive *in vivo* photoacoustic imaging of tumors. *Sci. Rep.* **6**, 33798; doi: 10.1038/srep33798 (2016).

## Supplementary Material

Supplementary Video S1

Supplementary Video S2

Supplementary Information

## Figures and Tables

**Figure 1:**
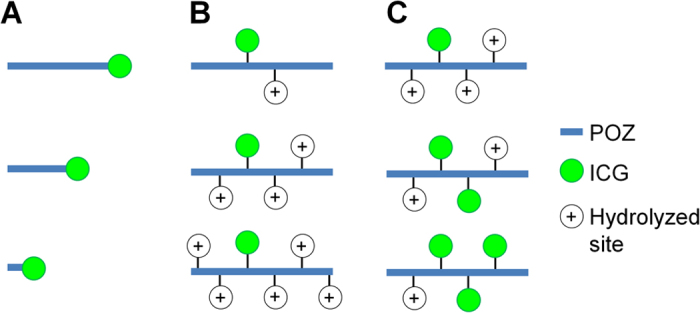
Drug design of POZ-ICG derivatives for sensitive PA tumor imaging in this study. We evaluated the influence of (**A**) molecular weight of POZ-ICG, (**B**) hydrolysis ratio in POZ-ICG, and (**C**) number of ICG molecules conjugated with POZ on probe accumulation in the tumor.

**Table 1:** Physicochemical properties of POZ-ICG derivatives having different molecular weights.

Sample	Molecular weight^a^ (kDa)	Number of ICG conjugated to POZ
P1-ICG1.1	7.7	1.1
P2-ICG1.1	12.4	1.1
P3-ICG0.9	15.8	0.9
P4-ICG0.7	26	0.7
P5-ICG0.8	31	0.8
P6-ICG1.0	50	1.0
P7-ICG1.1	200	1.1

^a^Molecular weight was determined by gel permeation chromatography (GPC) and expressed as weight-average molecular weight.

**Table 2:** Physicochemical properties and half-life in the blood of hydrolyzed POZ-ICG with different hydrolysis ratios.

Sample	Molecular weight^a^ (kDa)	Hydrolysis ratio (%)	p*K*a^b^	Electric conductivity^b^ (mS/cm)	Number of ICG molecules conjugated to POZ	Half-life in the blood^c^ (h)
P8-ICG1.5	25	2.5	10.1	3.9	1.5	4.9 ± 1.2
P9-ICG0.6	25	5	10.2	3.8	0.6	4.6 ± 0.6
P10-ICG0.6	25	10	10.2	3.4	0.6	3.8 ± 0.5
P11-ICG0.7	25	15	10.3	3.1	0.7	2.1 ± 0.4
P12-ICG1.9	25	20	10.2	2.9	1.9	1.1 ± 0.0
P13-ICG1.7	50	2.5	10.3	3.7	1.7	4.8 ± 1.2
P14-ICG0.5	50	5	10.2	3.4	0.5	4.5 ± 0.1
P15-ICG1.0	50	10	10.4	2.7	1.0	3.4 ± 0.4
P16-ICG1.1	50	15	10.3	2.4	1.1	2.0 ± 0.4
P17-ICG1.9	50	20	10.4	1.7	1.9	1.1 ± 0.2

^a^Molecular weight was determined by gel permeation chromatography (GPC) and expressed as weight-average molecular weight.

^b^p*K*a and electric conductivity were determined before ICG conjugation.

^c^Half-life of POZ-ICG in the blood was calculated based on probe concentration in blood collected from tumor-bearing mice.

**Table 3:** Physicochemical properties of POZ conjugated with multiple ICG molecules.

Sample	Molecular weight^a^ (kDa)	Hydrolysis ratio (%)	Reaction ratio of ICG/POZ	Number of ICG conjugated to POZ
P8-ICG0.7	25	2.5	1	0.7
P8-ICG1.5	25	2.5	3	1.5
P9-ICG0.6	25	5	2	0.6
P9-ICG2.6	25	5	10	2.6
P9-ICG5.4	25	5	20	5.4
P10-ICG0.6	25	10	2	0.6
P10-ICG4.4	25	10	10	4.4
P10-ICG8.6	25	10	20	8.6
P13-ICG1.7	50	2.5	3	1.7
P13-ICG2.8	50	2.5	10	2.8
P14-ICG0.5	50	5	2	0.5
P14-ICG1.9	50	5	10	1.9
P14-ICG4.7	50	5	20	4.7
P14-ICG7.8	50	5	40	7.8
P14-ICG10.3	50	5	300	10.3
P15-ICG1.0	50	10	2	1.0
P15-ICG3.6	50	10	10	3.6
P15-ICG9.0	50	10	20	9.0
P15-ICG10.7	50	10	40	10.7

^a^Molecular weight was determined by gel permeation chromatography (GPC) and expressed as weight-average molecular weight.

## References

[b1] Weissleder R. & Pittet M. J. Imaging in the era of molecular oncology. Nature 452, 580–589 (2008). 18385732 10.1038/nature06917 PMC2708079

[b2] Wang L. V. & Hu S. Photoacoustic tomography: *in vivo* imaging from organelles to organs. Science 335, 1458–1462 (2012). 22442475 10.1126/science.1216210 PMC3322413

[b3] Ntziachristos V. Going deeper than microscopy: the optical imaging frontier in biology. Nat. Methods 7, 603–614 (2010). 20676081 10.1038/nmeth.1483

[b4] Kanazaki K. . Development of anti-HER2 fragment antibody conjugated to iron oxide nanoparticles for *in vivo* HER2-targeted photoacoustic tumor imaging. Nanomedicine 11, 2051–2060 (2015). 26238078 10.1016/j.nano.2015.07.007

[b5] Moon H. . Amplified photoacoustic performance and enhanced photothermal stability of reduced graphene oxide coated gold nanorods for sensitive photoacoustic imaging. ACS Nano 9, 2711–2719 (2015). 25751167 10.1021/nn506516p

[b6] Onoe S., Temma T., Kanazaki K., Ono M. & Saji H. Development of photostabilized asymmetrical cyanine dyes for *in vivo* photoacoustic imaging of tumors. J. Biomed. Opt. 20, 096006 (2015). 26358819 10.1117/1.JBO.20.9.096006

[b7] Temma T., Onoe S., Kanazaki K., Ono M. & Saji H. Preclinical evaluation of a novel cyanine dye for tumor imaging with *in vivo* photoacoustic imaging. J. Biomed. Opt. 19, 090501 (2014). 25199057 10.1117/1.JBO.19.9.090501

[b8] Huang P. . Tumor-specific formation of enzyme-instructed supramolecular self-assemblies as cancer theranostics. ACS Nano 9, 9517–9527 (2015). 26301492 10.1021/acsnano.5b03874 PMC5223087

[b9] Cherrick G. R., Stein S. W., Leevy C. M. & Davidson C. S. Indocyanine green: observations on its physical properties, plasma decay, and hepatic extraction. J. Clin. Invest. 39, 592–600 (1960). 13809697 10.1172/JCI104072 PMC293343

[b10] de la Zerda A. . Family of enhanced photoacoustic imaging agents for high-sensitivity and multiplexing studies in living mice. ACS Nano 6, 4694–4701 (2012). 22607191 10.1021/nn204352r PMC3397693

[b11] Kanazaki K. . Development of human serum albumin conjugated with near-infrared dye for photoacoustic tumor imaging. J. Biomed. Opt. 19, 96002 (2014). 25191833 10.1117/1.JBO.19.9.096002

[b12] Kanazaki K. . Feasibility of poly(ethylene glycol) derivatives as diagnostic drug carriers for tumor imaging. J. Controlled Release 226, 115–123 (2016). 10.1016/j.jconrel.2016.02.017 26869546

[b13] Sano K. . *In vivo* photoacoustic imaging of cancer using indocyanine green-labeled monoclonal antibody targeting the epidermal growth factor receptor. Biochem. Biophys. Res. Commun. 464, 820–825 (2015). 26168727 10.1016/j.bbrc.2015.07.042

[b14] Maeda H. Tumor-selective delivery of macromolecular drugs *via* the EPR effect: background and future prospects. Bioconjug. Chem. 21, 797–802 (2010). 20397686 10.1021/bc100070g

[b15] Maeda H. & Matsumura Y. EPR effect based drug design and clinical outlook for enhanced cancer chemotherapy. Adv. Drug Delivery Rev. 63, 129–130 (2011). 10.1016/j.addr.2010.05.001 20457195

[b16] Viegas T. X. . Polyoxazoline: chemistry, properties, and applications in drug delivery. Bioconjug. Chem. 22, 976–986 (2011). 21452890 10.1021/bc200049d

[b17] Luxenhofer R. . Poly(2-oxazoline)s as polymer therapeutics. Macromol. Rapid Commun. 33, 1613–1631 (2012). 22865555 10.1002/marc.201200354 PMC3608391

[b18] Hoogenboom R., Wiesbrock F., Leenen M. A., Meier M. A. & Schubert U. S. Accelerating the living polymerization of 2-nonyl-2-oxazoline by implementing a microwave synthesizer into a high-throughput experimentation workflow. J. Comb. Chem. 7, 10–13 (2005). 15638473 10.1021/cc049846f

[b19] Huang H. . Solvent-induced morphological transition in core-cross-linked block copolymer micelles. J. Am. Chem. Soc. 128, 3784–3788 (2006). 16536553 10.1021/ja057762k

[b20] Gaertner F. C., Luxenhofer R., Blechert B., Jordan R. & Essler M. Synthesis, biodistribution and excretion of radiolabeled poly(2-alkyl-2-oxazoline)s. J. Controlled Release 119, 291–300 (2007). 10.1016/j.jconrel.2007.02.015 17451833

[b21] Obeid R. & Scholz C. Synthesis and self-assembly of well-defined poly(amino acid) end-capped poly(ethylene glycol) and poly(2-methyl-2-oxazoline). Biomacromolcules 12, 3797–3804 (2011). 10.1021/bm201048x PMC3197020 21875032

[b22] Gaspar V. M. . Poly(2-ethyl-2-oxazoline)-PLA-g-PEI amphiphilic triblock micelles for co-delivery of minicircle DNA and chemotherapeutics. J. Controlled Release 189, 90–104 (2014). 10.1016/j.jconrel.2014.06.040 24984013

[b23] Mero A. . Synthesis and characterization of poly(2-ethyl 2-oxazoline)-conjugates with proteins and drugs: suitable alternatives to PEG-conjugates? J. Controlled Release 125, 87–95 (2008). 10.1016/j.jconrel.2007.10.010 18031860

[b24] Tong J. . Conjugates of superoxide dismutase 1 with amphiphilic poly(2-oxazoline) block copolymers for enhanced brain delivery: synthesis, characterization and evaluation *in vitro* and *in vivo*. Mol. Pharmaceutics 10, 360–377 (2013). 10.1021/mp300496x PMC3570234 23163230

[b25] Brissault B. . Synthesis of linear polyethylenimine derivatives for DNA transfection. Bioconjugate Chem. 14, 581–587 (2003). 10.1021/bc0200529 12757382

[b26] Chujo Y., Sada K. & Saegusa T. Reversible gelation of polyoxazoline by means of Diels-Alder reaction. Macromolecules 23, 2636–2641 (1990).

[b27] Xiao K. . The effect of surface charge on *in vivo* biodistribution of PEG-oligocholic acid based micellar nanoparticles. Biomaterials 32, 3435–3446 (2011). 21295849 10.1016/j.biomaterials.2011.01.021 PMC3055170

[b28] Dellian M., Yuan F., Trubetskoy V. S., Torchilin V. P. & Jain R. K. Vascular permeability in a human tumour xenograft: molecular charge dependence. Br. J. Cancer 82, 1513–1518 (2000). 10789717 10.1054/bjoc.1999.1171 PMC2363402

[b29] Mordon S., Devoisselle J. M., Soulie-Begu S. & Desmettre T. Indocyanine green: physicochemical factors affecting its fluorescence *in vivo*. Microvasc. Res. 55, 146–152 (1998). 9521889 10.1006/mvre.1998.2068

[b30] Dragulescu-Andrasi A., Kothapalli S. R., Tikhomirov G. A., Rao J. & Gambhir S. S. Activatable oligomerizable imaging agents for photoacoustic imaging of furin-like activity in living subjects. J. Am. Chem. Soc. 135, 11015–11022 (2013). 23859847 10.1021/ja4010078 PMC3771329

[b31] Qin H., Zhou T., Yang S. & Xing D. Fluorescence quenching nanoprobes dedicated to *in vivo* photoacoustic imaging and high-efficient tumor therapy in deep-seated tissue. Small 11, 2675–2686 (2015). 25656695 10.1002/smll.201403395

[b32] Bai M. & Achilefu S. Synthesis and spectroscopy of near infrared fluorescent dyes for investigating dichromic fluorescence. Bioorg. Med. Chem. Lett. 21, 280–284 (2011). 21106373 10.1016/j.bmcl.2010.11.024 PMC3010286

[b33] Jo J., Nagane K., Yamamoto M. & Tabata Y. Effect of amine type on the expression of plasmid DNA by cationized dextran. J. Biomater. Sci., Polym. Ed. 21, 225–236 (2010). 20092686 10.1163/156856209X415549

