# Supplementary material for: Polyoxazoline multivalently conjugated with indocyanine green for sensitive in vivo photoacoustic imaging of tumors
Source: Sci Rep. 2016 Sep 26;6:33798. doi: 10.1038/srep33798 (PMC5036052; doi:10.1038/srep33798)
Supplement: Supplementary Information [file srep33798-s3.pdf]

Supplementary Information:

Polyoxazoline multivalently conjugated with indocyanine green for sensitive *in vivo* photoacoustic imaging of tumors

Kengo Kanazaki[1,2,¶], Kohei Sano[1,3,¶], Akira Makino[1,4], Tsutomu Homma[2], Masahiro Ono[1], Hideo Saji[1,*]

[1]Department of Patho-Functional Bioanalysis Graduate School of Pharmaceutical Sciences, Kyoto University, 46-29 Yoshida Shimoadachi-cho, Sakyo-ku, Kyoto, Japan, 606-8501

[2]Medical Imaging Project, Corporate R&D Headquarters, Canon Inc., 3-30-2 Shimomaruko, Ohta-ku, Tokyo, Japan, 146-8501

[3]Kyoto University Hospital, 54 Kawaharacho, Shogoin, Sakyo-ku, Kyoto, Japan, 606-8507

[4]Biomedical Imaging Research Center, University of Fukui, 23-3 Matsuokashimoaizuki, Eiheiji-cho, Yoshida-gun, Fukui, Japan, 910-1193

[*] Corresponding author

Hideo Saji, PhD, Professor

E-mail: hsaji@pharm.kyoto-u.ac.jp

[¶] These authors contributed equally to this work.

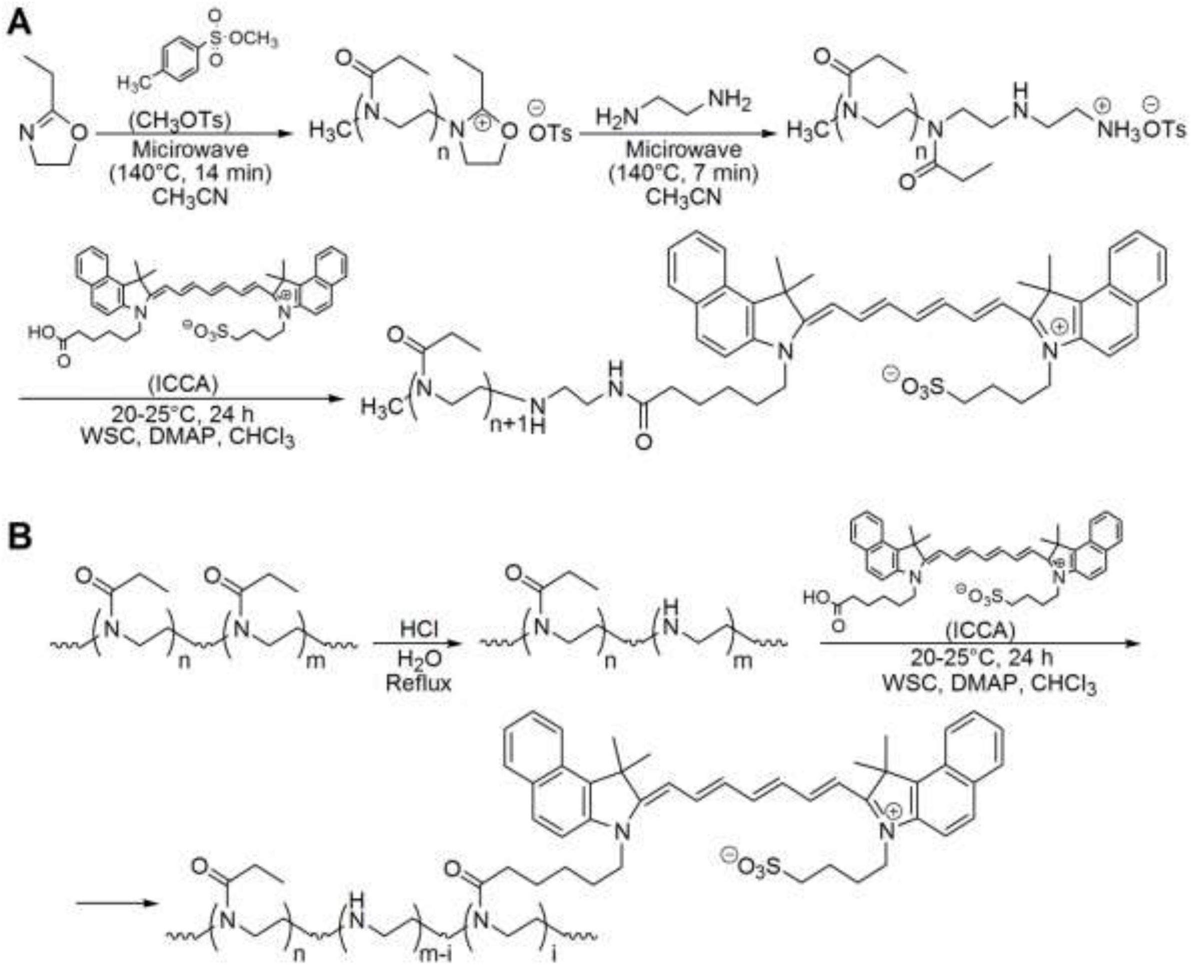

Figure S1. The synthesis scheme of POZ-ICG. (A) ICG was introduced into the terminal end of POZ derivatives synthesized *via* microwave irradiation. (B) Multiple ICG molecules were introduced into POZ derivatives whose acyl groups were partially hydrolyzed using hydrochloric acid.

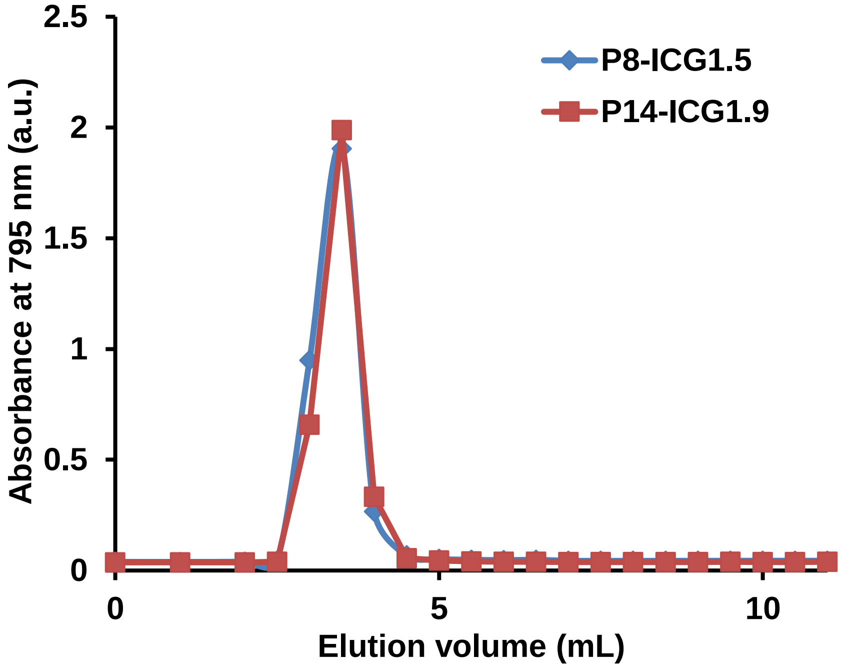

Figure S2. Size exclusion chromatography of POZ-ICG with PD-10 column. POZ-ICG was detected by absorbance at 795 nm. Representative charts of P8-ICG1.5 and P14-ICG1.9 were shown. The absorbance of ICG was detected only in high molecular weight fraction (3-4 mL).

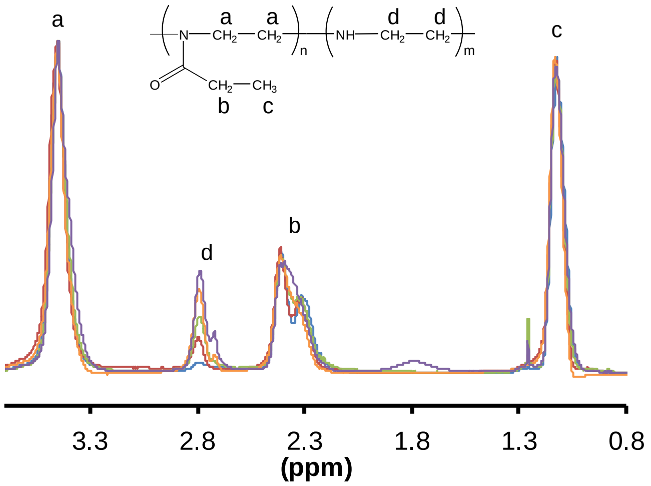

Figure S3. NMR spectrum of hydrolyzed POZ. Hydrolysis percentage of POZ was 2.5, 5, 10, 15, and 20% for blue, red, green, orange, and purple lines, respectively.

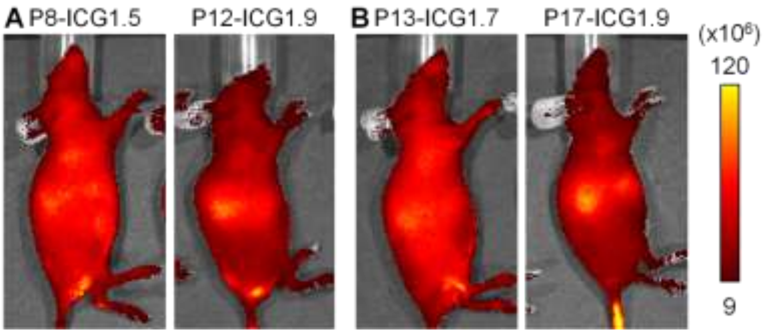

Figure S4. *In vivo* fluorescent images of tumor-bearing mice at 1 h after administration with POZ-ICG (A; 25 kDa, B; 50 kDa). (A) Left: P8-ICG1.5, right: P12-ICG1.9. (B) Left: P13-ICG1.7, right: P17-ICG1.9 as shown in Table 2. Scale bar units: photons/sec/cm^2/steradian.

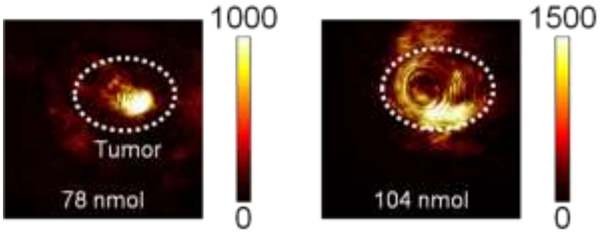

Figure S5. Adjusted *In vivo* PA images of tumor-bearing mice at 24 h after administration with POZ-ICG (left: 78 nmol, right: 104 nmol). The color dynamic range in Fig. 5B was adjusted so as to prevent PA signal saturation. Dotted circles indicated the tumor regions. Scale bar units: arbitrary unit.

Table S1. *In vivo* biodistribution data of POZ conjugated with multiple ICG molecules.

Sample	Hydrolysis ratio (%)	Number of ICG conjugated with POZ	Tumor accumulation (%ID/g)	Probe concentration in the blood (%ID/g))	Tumor-to-blood ratio
P8-ICG0.7	2.5	0.7	7.7±0.2	2.1±0.5	3.9±0.9
P8-ICG1.5	2.5	1.5	8.7±1.2	0.9±0.1	9.2±2.0
P9-ICG0.6	5	0.6	7.9±0.5	1.4±0.2	5.5±0.3
P9-ICG2.6	5	2.6	5.8±0.9	0.4±0.1	15.4±2.4
P9-ICG5.4	5	5.4	3.7±0.2	0.3±0.0	11.3±0.8
P10-ICG0.6	10	0.6	7.5±0.3	0.5±0.1	14.1±2.1
P10-ICG4.4	10	4.4	2.8±0.2	0.1±0.0	22.7±3.9
P10-ICG8.6	10	8.6	1.6±0.1	0.2±0.0	11.0±3.1
P13-ICG1.7	2.5	1.7	7.2±0.5	3.6±0.3	2.8±0.3
P13-ICG2.8	2.5	2.8	8.2±1.8	2.9±0.5	2.9±0.7
P14-ICG0.5	5	0.5	7.6±1.1	2.6±0.2	2.9±0.5
P14-ICG1.9	5	1.9	6.6±1.9	1.8±0.3	3.6±0.5
P14-ICG4.7	5	4.7	9.5±1.4	2.0±0.3	4.8±1.2
P14-ICG7.8	5	7.8	9.4±0.6	1.5±0.1	6.3±1.0
P14-ICG10.3	5	10.3	4.1±0.5	0.6±0.1	7.6±0.9
P15-ICG1.0	10	1.0	7.4±1.2	1.6±0.1	4.7±0.6
P15-ICG3.6	10	3.6	4.7±0.7	0.5±0.1	8.7±0.4
P15-ICG9.0	10	9.0	3.0±0.3	0.1±0.1	23.4±2.2
P15-ICG10.7	10	10.7	3.5±0.6	0.2±0.1	23.5±7.8

Video S1. *In vivo* 3D PA image before POZ-ICG administration (also shown in Fig. 5B).

Video S2. *In vivo* 3D PA image 24 h after POZ-ICG administration (52 mol) (also shown in Fig. 5C).
